# Case report: A successful re-challenge report of GLS-010 (Zimberelimab), a novel fully humanized mAb to PD-1, in a case of recurrent endometrial cancer

**DOI:** 10.3389/fimmu.2022.987345

**Published:** 2022-10-06

**Authors:** Yeshan Chen, Ai Huang, Qin Yang, Jing Yu, Guiling Li

**Affiliations:** ^1^ Cancer Center, Union Hospital, Tongji Medical College, Huazhong University of Science and Technology, Wuhan, China; ^2^ Department of Radiation and Medical Oncology, Hubei Key Laboratory of Tumor Biological Behaviors, Hubei Cancer Clinical Study Center, Zhongnan Hospital of Wuhan University, Wuhan, China

**Keywords:** GLS-010, re-challenge, immune checkpoint inhibitors, endometrial cancer, myocarditis

## Abstract

With the widespread use of immune checkpoint inhibitors (ICI), there is growing concern about reports of immune-related adverse events (irAE). In clinical practice, patients who experience severe toxicities by ICI-based therapies would require utmost caution in resuming ICI therapy because of the potential risk of serious irAEs caused by the reintroduction of immunotherapy. In this study, we report a case of recurrent endometrial cancer patient with PD-L1 positive as well as dMMR suffering from immunotherapy-associated myocarditis after first-line treatment with ICI combined with a multi-targeted anti-angiogenic agent. After symptomatic treatment, the patient was in complete remission from treatment toxicities. Subsequently, through MDT discussions, we selected a new PD-1 agent, zimberelimab, for rechallenge therapy, and the patient achieved a sustained disease remission without any treatment-related toxicities. To date, the manner and timing of the ICI re-challenge has been a subject of iterative deliberation. We believe that our experience could shed some light on ICI rechallenge therapy, and we look forward to more literatures to refine the ICI rechallenge scenarios.

## Introduction

With the introduction of immune checkpoint inhibitors (ICI) such as programmed cell death-1 (PD-1) and programmed cell death-ligand 1 (PD-L1) antibodies, a significant improvement of the efficacy was seen in numerous malignancies (1–3). Supported by a growing body of evidence, ICI-based therapies have gradually crossed over from backline to frontline treatments (4, 5) and even emerged in the neoadjuvant front (6). In the context of widespread use of ICIs, the number of patients developing immune-related adverse events (irAE) is also rising (7). The category and grading of irAE can be attributed to a variety of factors, including the type of ICIs, the mode of administration such as monotherapy or in combined with chemo- or radiotherapy, and the duration of ICI use, etc.

Myocarditis is a very rare but highly lethal irAE, with the reported incidence of less than 1% (8). According to irAE management guidelines, once myocarditis has occurred, restarting ICI therapy requires great caution even after complete resolution of symptoms, and the manner and timing of ICI resumption is still an open issue. Herein, we would share a case of endometrial cancer who had developed immune-associated myocarditis and was successfully treated with a re-challenge of PD-1.

## Case report

A 58-year-old female patient was admitted with “intermittent vaginal bleeding for 1 month”. After admission, the patient was diagnosed with endometrial cancer by hysteroscopy. On December 9, 2020, she underwent a total hysterectomy combined with pelvic and para-aortic lymph node dissection. The postoperative pathological diagnosis showed low-differentiated endometrioid adenocarcinoma, invading the outer 1/2 layer of the uterine wall, with a large number of vascular carcinoma thrombi visible, no cancerous tissue involved in the cervix and parametrium area, 1/13 metastases in the para-aortic lymph nodes and 1/33 metastases in the pelvic lymph nodes, the combined positive score (CPS) for PD-L1 (22C3) was 2 and the tumor presented as dMMR. Since January 2021, the patient received 3 cycles of adjuvant chemotherapy with liposomal paclitaxel (135 mg/m^2^, iv. Q3w) in combination with carboplatin (AUC = 5, iv. Q3w), during which, after 2 cycles of chemotherapy, she underwent an adjuvant radiotherapy to the pelvic and para-aortic lymph node areas at a dose of 50.4Gy in 28 fractions. On May 12, 2021, she underwent a routine computed tomography (CT) review showing abnormally enlarged left parietal abdominal aortic lymph nodes and diagnosed as tumor progression. On May 21, 2021, she was enrolled in a clinical study of Fruqintinib in combination with Sintilimab for advanced solid tumors (registry number: CTR20190514), in which Fruquintinib (5 mg, po. d1-21, Q4w), and Sintilimab (200 mg, iv. Q3w) for 2 cycles. Fortunately, the tumor was in partial remission at the end of 2 cycles of treatment by routine review.

**Figure 1:**
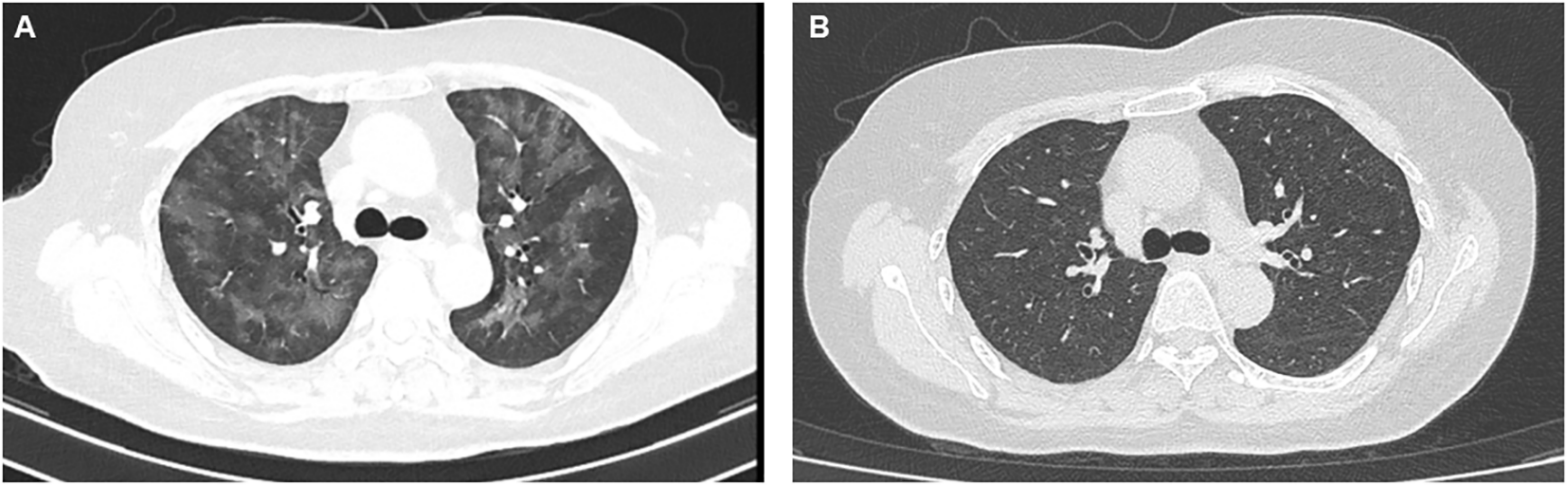
The performance of CT scan of the pulmonary infection induced by hormone use **(A)** and the result of CT image after anti-infection as well as symptomatic treatment **(B)**.

However, on June 30, 2021, the patient began complaining of shortness of breath after mild activity and abnormally elevated blood troponin up to 250 ng/L, along with the abnormal elevation of creatine kinase (up to 432 U/L) and lactate dehydrogenase (up to 325 U/L). No significant abnormalities were seen on the electrocardiogram, and she was diagnosed with grade 2 ICI-associated myocarditis. ICI therapy was immediately suspended and symptomatic treatment with methylprednisolone was administered. On September 2, 2020, the patient complained of increased chest tightness and shortness of breath, and blood troponin showed 47.3 ng/L. Chest CT reported interstitial pneumonia in both lungs (**Figure 1A**), and genetic testing of pathogenic microorganisms from alveolar lavage fluid was diagnosed as secondary *Pneumocystis jirovecii* infection due to immune depression. Targeted anti-infective therapy was given, along with symptomatic treatment with methylprednisolone combined with human immunoglobulin. Until November 2, 2021, the patient’s shortness of breath gradually improved, and the chest CT showed that the inflammatory manifestations of the lungs basically disappeared (**Figure 1B**), and the myocardial enzyme index returned to the normal range. However, unfortunately, due to the interruption of antitumor treatment for 5 months, the para-aortic lymph nodes were significantly enlarged and fuses into clusters, which was considered as tumor progression.

**Figure 2:**
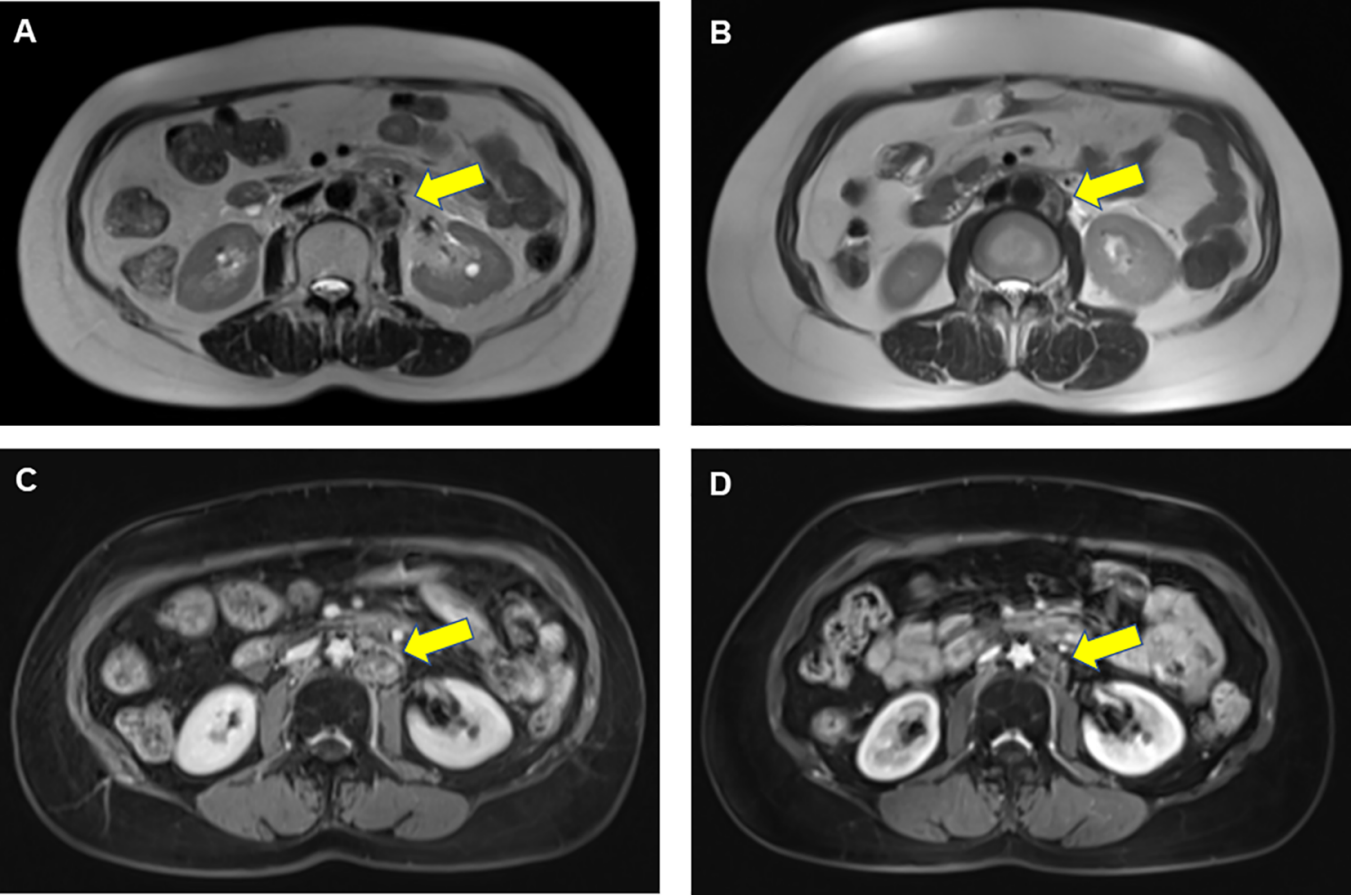
Results of abdominal MRI scans before and after ICI rechallenge treatment in a patient with endometrial cancer. Prior to PD-1 rechallenge, T2-weighted image **(A)** and T1-weighted enhanced scans **(C)** showed enlarged lymph nodes visible next to the abdominal aorta. After 2 cycles of PD-1 rechallenge, T2-weighted image **(B)** and T1-weighted enhanced sequence **(D)** indicated that those lymph nodes achieved partial remission. The yellow arrow represented tumor locations.

**Figure 3:**
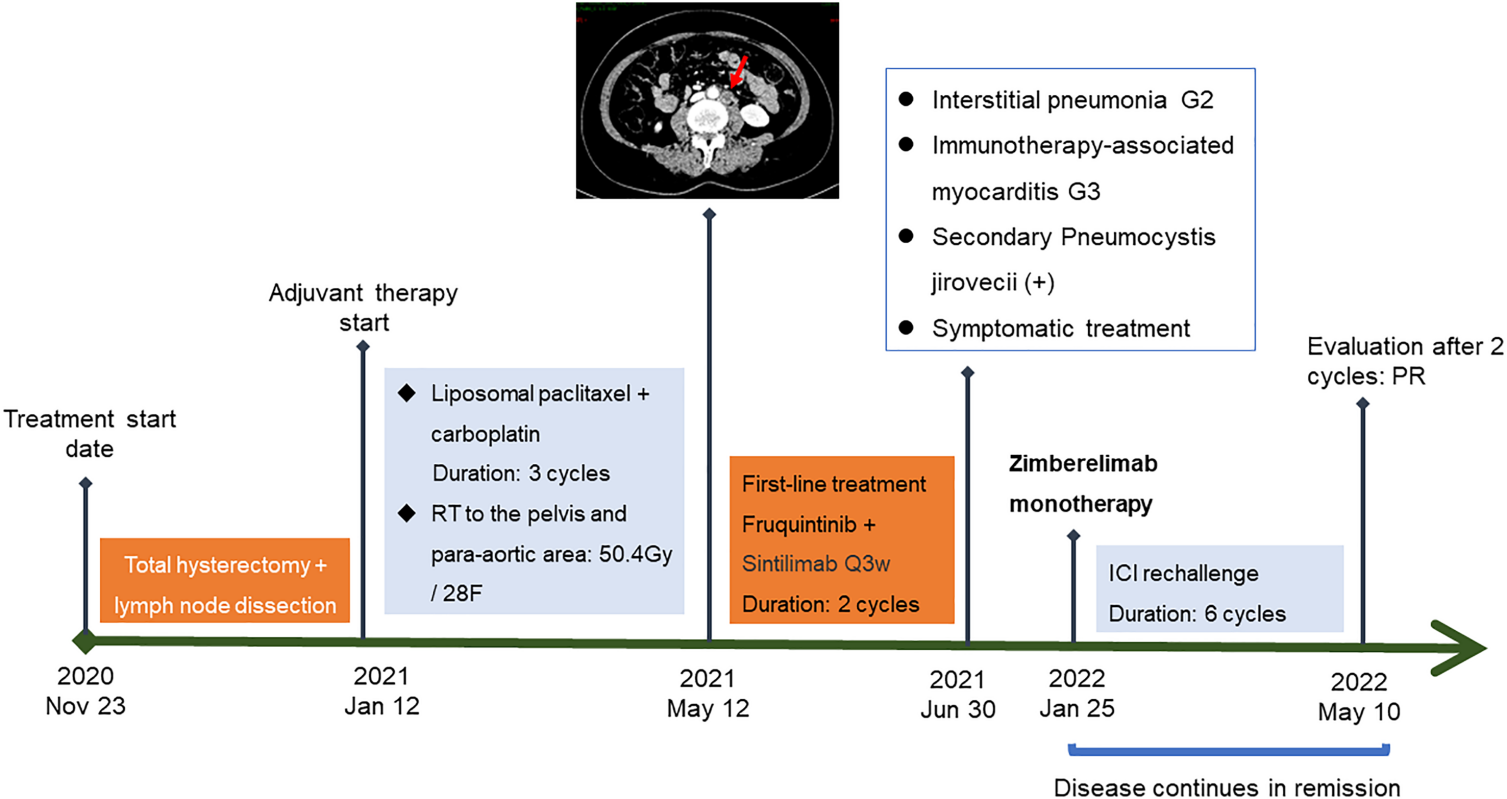
The entire treatment history of a patient with recurrent endometrial cancer treated with PD-1 rechallenge.

Given the benefit of previous immunotherapy, after a multi-disciplinary treatment (MDT) discussion and a thorough risk communication with the patient, she began treatment with Zimberelimab (240 mg, iv. Q3w), a novel fully humanized mAb to PD-1, from January 25, 2022, the tumor was in partial remission after 2 cycles of treatments (**Figure 2**), and the disease remained in continuous remission until our latest follow-up visit, September 14, 2022. Surprisingly, the patient did not experience any elevation of cardiac enzymes or symptoms related to myocarditis throughout the immunotherapy. The entire treatment history could be seen in **Figure 3**.

## Discussion

Based on the published experience, for those who had severe irAEs, the restart of ICI therapy can be implemented in three main scenarios: first, by switching from anti-PD-(L)1 antibodies to anti-CTLA-4 approach or vice versa, which must be initiated on the premise that both types of ICI have a definite efficacy in that disease; second, by choosing the same class of ICI but with a different agent when irAEs have largely resolved; and third, the restarting ICI should be under conditions where a secondary prevention efforts has been well-established (9).

In this case, by a multidisciplinary discussion, we chose to restart ICI therapy in the second script. A retrospective study that adopted the same class of ICI re-challenge method reported that 40 (50%) of the 80 patients would reoccur with varying degrees or types of toxicities, but only 14 (18%) cases would experience a recurrence of those initial irAEs (10). Another retrospective study including 38 NSCLCs showed that by the same type of ICI treatment, 18 (48%) patients would not experience any further irAEs and 10 (26%) patients developed new irAEs, compared with only 10 (26%) of the initial irAEs. These recurrent and new irAEs were mild and manageable (11). Accordingly, it is worthwhile to consider similar PD-(L)1 rechallenge therapy for this patient. Through 6 cycles of treatment, her disease achieved a well control. More importantly, she did not experience any further irAEs.

Targeting inhibition of vascular endothelial growth factor (VEGF)/VEGF receptor 2 (VEGFR2) could not only reduce tumor growth but also improve the vascular normalization and modulate the response to immunotherapy (12). Although the patient suffered from the immune myocarditis after first-line Fruqintinib/Sintilimab treatment, we could not deny that the modulation of the immune microenvironment by Fruqintinib underlined her long survival benefit.

It has been reported that cardiovascular toxicity is one of the major toxicities of anti-angiogenic drugs (13). However, published literature showed that the most common toxicities of the multitargeted agent, Fruqintinib, were hypertension, coagulation disorders, and thrombosis. Elevated cardiac enzymes were a very rare event. Therefore, we hypothesized that the myocardial injury in this case was largely attributed to ICI-related toxicity.

To summarize, our courage to re-challenge ICI was based on the following conditions. Her postoperative pathological results showed tumor was positive PD-L1 expression and dMMR status, which strongly predicted that she would most likely benefit from PD-1 therapy (14, 15), and previous literatures showed that the risk of initial irAEs after re-challenge treatment was not irreversible and those initial toxicities did not necessarily return. Then, Zimberelimab, as a new PD-1 antibody, has not been reported any cardiovascular events based on the current evidence. In addition, there are no superior later line options for her after failure from first-line therapy. In light of the recent study, Zimberelimab has demonstrated favorable preliminary results in the recurrent gynecologic malignancies (16). Finally, on the basis of MDT, we reduced the dose intensity of Zimberelimab (from 240mg Q2w to 240 mg Q3w) to possibly maximize the safety of the restart ICI treatment.

## Patient perspective

Although ICIs-associated myocarditis is an uncommon event, it is a highly lethal toxicity. According to retrospective studies, the probability of recurrence of initial toxicities after ICIs re-challenge was less than 30%. It was confirmed by our case report that, under the framework of MDT, PD-1 re-challenge would be feasible and manageable for those who are potential benefit from ICIs.

## Data availability statement

The raw data supporting the conclusions of this article will be made available by the authors, without undue reservation.

## Ethics statement

Written informed consent was obtained from the individual(s) for the publication of any potentially identifiable images or data included in this article.

## Author contributions

YC and JY designed and drafted the manuscript. AH, QY and GL providing suggestions for revisions and review of the manuscript. All authors contributed to the article and approved the submitted version.

## Conflict of interest

The authors declare that the research was conducted in the absence of any commercial or financial relationships that could be construed as a potential conflict of interest.

## Publisher’s note

All claims expressed in this article are solely those of the authors and do not necessarily represent those of their affiliated organizations, or those of the publisher, the editors and the reviewers. Any product that may be evaluated in this article, or claim that may be made by its manufacturer, is not guaranteed or endorsed by the publisher.
